# Whole Transcriptomic Analysis Identifies Candidate Biomarkers from Saliva of Temporomandibular Joint Osteoarthritis Patients

**DOI:** 10.3390/ijms27062727

**Published:** 2026-03-17

**Authors:** Nawal Alketbi, Alaa Muayad Altaie, Reem Sami Alhamidi, Ayesha Yusuf Phansupkar, Alaa Mohamed Hamad, Mohamed Haider, Rania Harati, Kathrin Kalies, Wael Talaat, Rifat Hamoudi

**Affiliations:** 1 Department of Oral and Craniofacial Health Sciences, College of Dental Medicine, University of Sharjah, Sharjah P.O. Box 27272, United Arab Emirates; wtaha@sharjah.ac.ae; 2 Research Institute for Medical and Health Sciences, University of Sharjah, Sharjah P.O. Box 27272, United Arab Emirates; 3 Center of Excellence for Precision Medicine, University of Sharjah, Sharjah P.O. Box 27272, United Arab Emirates; 4 College of Health Sciences, Abu Dhabi University, Abu Dhabi P.O. Box 59911, United Arab Emirates; 5 Department of Pharmaceutics and Pharmaceutical Technology, College of Pharmacy, University of Sharjah, Sharjah P.O. Box 27272, United Arab Emirates; 6 Department of Pharmacy Practice and Pharmacotherapeutics, College of Pharmacy, University of Sharjah, Sharjah P.O. Box 27272, United Arab Emirates; 7 Institute of Anatomy, University of Luebeck, 23562 Luebeck, Germany; 8 Department of Clinical Sciences, College of Medicine, University of Sharjah, Sharjah P.O. Box 27272, United Arab Emirates; 9 Division of Surgery and Interventional Science, University College London, London WC1E 6TB, UK; 10 Biomedically Informed Artificial Intelligence Laboratory (BIMAI-Lab), University of Sharjah, Sharjah P.O. Box 27272, United Arab Emirates

**Keywords:** temporomandibular osteoarthritis, saliva, biomarkers, transcriptomics, RNA sequencing, *CRIP1*, *PPA1*, *TARS1*, *GCLC*

## Abstract

Temporomandibular joint osteoarthritis (TMJOA) is a degenerative disease characterized by progressive cartilage degeneration and subchondral bone remodeling, resulting in chronic pain and functional impairment. Although conservative treatments such as physical therapy and non-steroidal anti-inflammatory drugs (NSAIDs) are commonly used, their effectiveness is limited due to the poorly understood pathophysiology of TMJOA. Identifying reliable molecular biomarkers is essential to improving early diagnosis and guiding therapeutic development. This proof-of-concept study aims to identify candidate salivary biomarkers for TMJOA using an integrative approach combining clinical validation with in silico analysis. RNA sequencing was performed on saliva samples from TMJOA patients and healthy controls. In parallel, publicly available transcriptomic dataset GSE205389 was analyzed to identify differentially expressed genes (DEGs). DEGs were validated using qRT-PCR. Gene set enrichment analysis (GSEA) and Metascape were used to explore biological pathways associated with TMJOA. Integration of clinical and in silico RNA sequencing datasets identified 2758 and 3548 DEGs, respectively, with 743 overlapping genes. Pathway enrichment analyses highlighted immune-related, metabolic and osteoclast-related pathways. Four genes, *CRIP1*, *PPA1* and *TARS1* (statistically significant) and *GCLC* (non-significant trend), were validated by qRT-PCR in the clinical saliva samples, confirming elevated expression in TMJOA patients. Validation of the in silico dataset showed an upregulation of *PTK2B*, *ABL1*, *TNF* and *IL-1B,* supporting their relevance as salivary biomarkers in TMJOA. This exploratory study identifies four candidate salivary genes, *CRIP1*, *PPA1*, *TARS1* and *GCLC*, as candidate salivary biomarkers for TMJOA, offering insights into disease mechanisms. Larger studies are needed to validate these findings and assess their clinical utility.

## 1. Introduction

Temporomandibular joint (TMJ) osteoarthritis (TMJOA) is a chronic degenerative disease characterized by progressive degradation of the articular cartilage and remodeling of the subchondral bone, resulting in severe pain and functional impairment in the TMJ [1]. Various factors, including age, gender, mechanical stress, oral parafunctional behavior and psychological stress, have been associated with its etiology [2]. Conservative treatments such as physical therapy, occlusal splints, non-steroidal anti-inflammatory drugs (NSAIDs), as well as minimally invasive surgeries like arthrocentesis and arthroscopy, remain the major therapeutic approach for TMJOA in clinical practice [3]. However, these treatments often fail to provide long-term relief, primarily due to the poorly understood pathophysiology of the disease [4].

Articular cartilage functions in a metabolically active environment with limited oxygen and glucose availability, making it highly susceptible to metabolic stress [5]. Under pathological conditions, cellular metabolism becomes dysregulated, leading to increased production of pro-inflammatory, pro-catabolic and anti-anabolic factors [6]. Various biological fluids, including synovial fluid, blood, plasma and saliva, have been explored as diagnostic sources of inflammatory mediators contributing to TMJOA. Although synovial fluid directly reflects the joint microenvironment, its collection is invasive and not suitable for routine clinical screening or longitudinal monitoring [7]. Circulating blood-based biomarkers may be affected by systemic dilution and confounding inflammatory conditions [8,9]. In contrast, saliva represents a readily accessible and non-invasive biofluid that captures systemic inflammatory and metabolic signals through diffusion from blood and local immune activity within the oral cavity [10,11,12,13,14]. Increasing evidence supports the use of salivary molecular biomarkers in inflammatory and musculoskeletal disorders, highlighting saliva as a clinically feasible medium for early disease detection and monitoring [10,11,12,13,15,16]. Therefore, despite the anatomical distinction between the TMJ and oral cavity, saliva provides an integrative biological snapshot relevant to TMJOA pathophysiology [10]. A systematic review of eleven clinical studies found strong correlations between salivary biomarkers (cortisol, *IL-1*, and glutamate) and temporomandibular disorders (TMD). Elevated cortisol and *IL-1* levels suggest a possible role of stress and inflammation in disease progression, while increased glutamate levels may be associated with pain signaling [10].

High-throughput computational transcriptomic approaches have become a powerful approach for exploring the molecular and biomechanical mechanisms underlying TMJOA. Transcriptomic studies using RNA sequencing (RNA-seq) have identified differentially expressed genes (DEGs) and dysregulated pathways related to inflammation, extracellular matrix degradation and oxidative stress, all contributing to TMJOA progression. Integrating in silico analysis techniques with multi-omics data enhances our understanding of TMJOA at both molecular and biomechanical levels and facilitates the discovery of novel biomarkers needed for early diagnosis. Indeed, early detection is crucial to halt disease progression in its initial stages, and identifying novel therapeutic targets remains essential to improving treatment outcomes for TMJOA patients. This study was designed as an exploratory proof-of-concept investigation to identify candidate salivary transcriptomic biomarkers associated with TMJOA using an integrative strategy combining clinical validation with in silico analysis.

## 2. Results

### 2.1. Gene Set Enrichment Analysis Identified Differentially Expressed Genes

**Figure 1:**
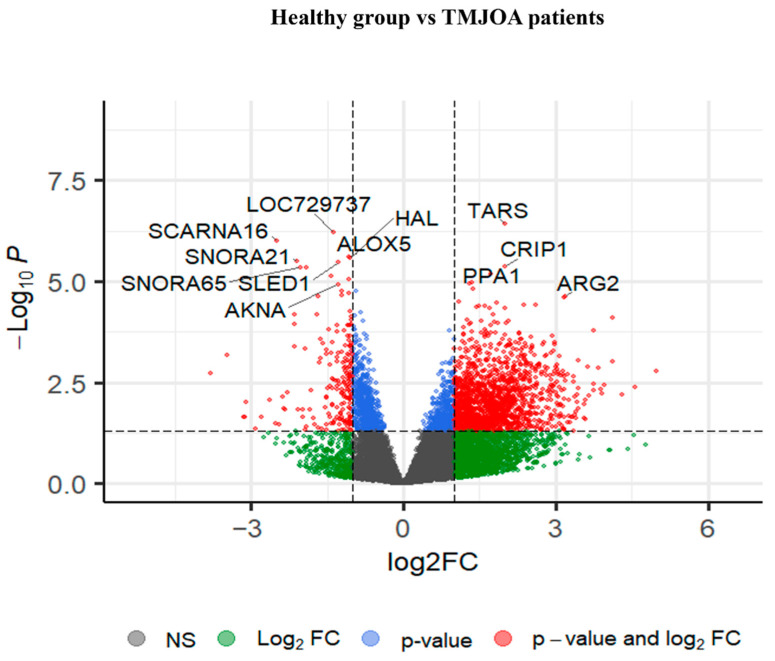
Volcano plot of differentially expressed genes between TMJOA patients and healthy controls (GSE289871). Volcano plot illustrating the differentially expressed genes that were identified from the RNA sequencing of saliva samples (GSE289871). The x-axis represents the log_2_ fold change (log_2_FC) in gene expression, while the y-axis represents the −log_10_ of the *p*-value. Red dots indicate genes with both statistically significant fold change (|log_2_FC| > 1.5) and *p*-value < 0.05. Blue dots represent genes with significant *p*-values but not fold changes, while green dots represent genes with significant fold changes but non-significant *p*-values.

Differential expression analysis was performed using DESeq2 on both our clinical RNA-seq dataset (GSE289871) and the in silico dataset (GSE205389). This resulted in the identification of 2758 DEGs (nominal *p* < 0.05) in the clinical cohort and 3548 DEGs in the in silico dataset. Figure 1 presents a volcano plot generated from the GSE289871 clinical dataset, displaying the distribution of DEGs between TMJOA patients and healthy controls. Genes with both statistical significance (nominal *p* < 0.05) and a log_2_ fold change >±1.5 are shown in red. Notably upregulated genes in TMJOA include *CRIP1*, *PPA1*, *TARS1* and *ARG2*, while downregulated genes include *SCARNA16*, *SNORA21*, *ALOX5* and *AKNA*.

### 2.2. Gene Set Enrichment Analysis Highlights TMJOA-Specific Activated Cellular Pathways

Absolute GSEA was conducted on the DEGs from both datasets, GSE289871 (clinical cohort) and GSE205389 (in silico dataset), using the C5 (Gene Ontology Biological Process) and C7 (Immunologic Signature) gene sets. The aim was to identify differentially active pathways between the healthy control and TMJOA groups using the false discovery rate (FDR) < 0.25 and *p*-value < 0.05 as statistical thresholds.

The clinical dataset (GSE289871) yielded 23 enriched pathways, including seven from C5 and 16 from C7 (Supplementary Table S3). The in silico dataset (GSE205389) revealed a total of 77 significantly enriched pathways: 73 from the C5 collection and four from the C7 collection (Supplementary Table S4).

In the clinical cohort (GSE289871), enrichment analysis of the C5 gene set revealed a significant activation of metabolic and stress-related biological processes, such as amino acid metabolic process, alpha-amino acid metabolic process and heat shock protein binding. These findings suggest that TMJOA in the clinical population may involve metabolic dysregulation and cell stress responses.

The in silico analysis highlighted several biologically relevant and significantly enriched pathways, such as actin-filament-based process, actin-filament polymerization, regulation of osteoclast differentiation and germinal center B-cell differentiation. These pathways may play critical roles in TMJOA pathogenesis.

**Figure 2:**
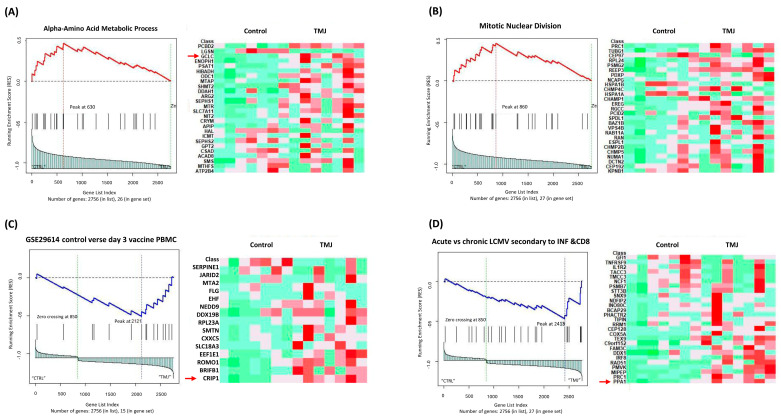
Representative GSEA-enriched pathways from the clinical RNA-seq dataset (GSE289871). Panels (**A**,**B**) show enriched pathways from the C5 (Gene Ontology) collection, including alpha-amino acid metabolic process and mitotic nuclear division, while panels (**C**,**D**) highlight pathways from the C7 (immunologic signature) collection. The enrichment plots (left panels) show the running enrichment score across the ranked gene list. Heatmaps (right panels) illustrate gene expression levels, with red indicating higher expression and green indicating lower expression. Genes boxed on the right were prioritized for validation based on biological relevance and frequency across multiple pathways. Healthy controls (CTRL) and TMJOA patients (TMJ) are indicated across all panels.

**Figure 3:**
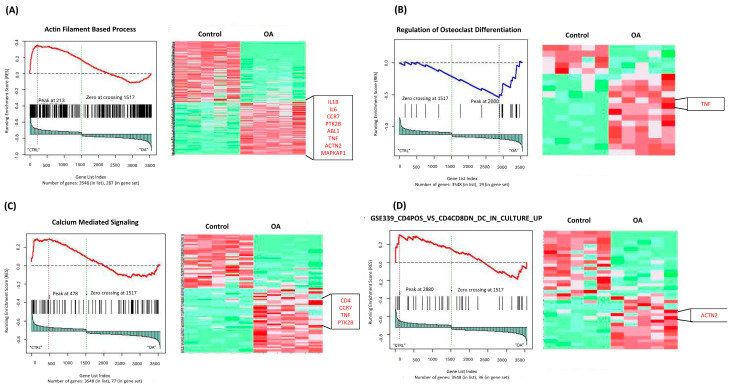
(**A**–**D**) Representative GSEA-enriched pathways from the in silico RNA-seq dataset (GSE205389). Heatmaps illustrate gene expression changes, with red indicating higher expression and green indicating lower expression. Genes highlighted in the red boxes were selected for downstream validation based on their biological relevance and frequency across enriched pathways.

Figure 2 and Figure 3 show representative GSEA-enriched pathways from the clinical (GSE289871) and in silico (GSE205389) datasets, respectively.

### 2.3. GSEA Identifies B-Cells Immunological Signature as One of the Activated Pathways Altered in TMJOA

GSEA using the C7 immunologic signature collection was performed on both the clinical RNA-seq dataset (GSE289871) and the in silico dataset (GSE205389) to explore immune-related transcriptomic changes in TMJOA. In the clinical dataset, several pathways related to B-cell activation and adaptive immune responses were significantly enriched, suggesting a potential role for these immune cells in TMJOA development. Other pathways indicated broader immune involvement, including responses typically triggered by infections and inflammation (Figure 2, Supplementary Table S3).

Similarly, the in silico dataset also revealed the enrichment of immune-related pathways. These included signatures related to innate immunity, T-cell regulation, and immune cell differentiation, all pointing to an altered immune landscape in TMJOA patients compared to healthy individuals (Figure 3, Supplementary Table S4).

Together, the findings from both datasets suggest that TMJOA may involve complex immune dysregulation, with contributions from both innate and adaptive immunity. The presence of B-cell–related and inflammatory signatures supports the idea that the immune system plays a central role in disease progression.

To prioritize the genes that were most relevant to the disease-related processes, a frequency analysis was performed on the genes appearing in the enriched pathways. Genes occurring in more than three distinct pathways were considered frequent. From each dataset, the top 20 most frequently occurring genes were selected for further investigation. Supplementary Table S5 presents the top 20 most frequent genes that were identified from the GSEA of the two datasets.

### 2.4. Common Genes and Enriched Pathways Identified in Both RNA-seq and In Silico Analyses

**Figure 4:**
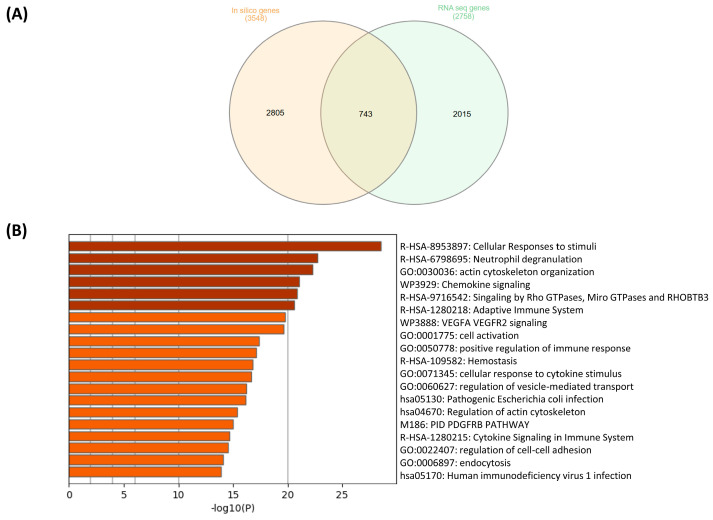
Overlap of genes and enriched pathways identified from the clinical RNA sequencing dataset (GSE289871) and the in silico dataset (GSE205389). (**A**) Venn diagram showing the overlap of differentially expressed genes (DEGs) between the in silico dataset (GSE205389) and the RNA sequencing data from the clinical saliva cohort (GSE289871), with 743 common DEGs. (**B**) Bar graph showing the top enriched biological pathways associated with the shared DEGs, based on Metascape analysis. Enriched pathways include immune response, chemokine signaling, actin cytoskeleton organization and cellular response to stress, suggesting a potential role in TMJOA pathogenesis.

To identify robust gene signatures associated with TMJOA, we compared the differentially expressed genes (DEGs) identified from both the clinical RNA sequencing dataset (GSE289871) and the in silico dataset (GSE205389). A total of 743 DEGs were found to be shared between the two datasets, as shown in Figure 4A.

Among these, 16 genes were consistently elevated in both datasets and appeared frequently across enriched pathways from the C5 (Gene Ontology) collection. These included genes such as *GCLC*, *SHMT2*, *DDAH1*, *HSPA1A*, *ALDH1A1*, *HSPA8* and *CRIP1*, many of which are associated with oxidative stress response, cellular metabolism and protein folding biological processes known to be relevant in joint degeneration and inflammation. In the C7 (immunologic signature) dataset, two genes, *ODC1* and *ALDH1A1*, were commonly identified in both analyses, suggesting potential roles in immune modulation within TMJOA pathology.

Functional enrichment analysis of the shared DEGs using Metascape revealed convergence on several key biological processes, including cellular response to stimuli, neutrophil degranulation, actin cytoskeleton organization and chemokine signaling pathways (Figure 4B). These pathways are particularly relevant to TMJOA, as they reflect inflammatory mechanisms, cytoskeletal remodeling and cellular stress responses that may contribute to disease progression and joint tissue dysfunction.

### 2.5. Experimental Validation of the Candidate Biomarkers Using qRT-PCR in Clinical Samples

To validate the transcriptomic findings, quantitative real-time PCR (qRT-PCR) was performed on the clinical saliva samples.

Saliva is an established liquid biopsy that captures circulating systemic molecular information, rather than reflecting only local site-specific pathology. It is widely used as a reliable non-invasive source of nucleic acids for disease risk stratification and biomarker discovery, even in conditions not anatomically related to the oral cavity. For instance, a large UK-based study demonstrated that saliva-derived DNA can be used to calculate a polygenic risk score for prostate cancer, enabling the effective screening of asymptomatic individuals. Notably, individuals in the highest risk group showed a cancer detection rate of approximately 40%, with more than 55% presenting a clinically significant disease, highlighting the ability of salivary nucleic acids to capture biologically meaningful systemic disease signals [17]. In another study, saliva was successfully used as a non-invasive medium to detect elevated amphiregulin in mRNA and protein levels in asthmatic patients, reflecting airway inflammation and correlating with disease severity and clinical parameters [18].

**Figure 5:**
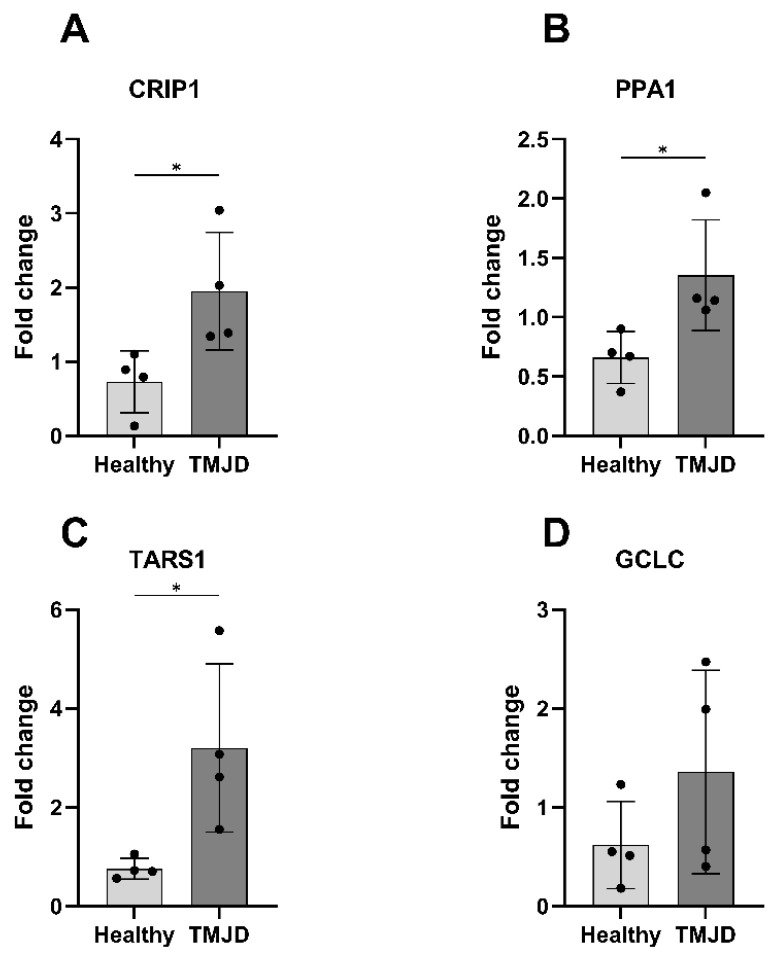
qRT-PCR validation of RNA-seq-identified genes in the clinical saliva cohort (GSE289871). Bar graphs showing the fold change expression of four genes in saliva samples from healthy controls and TMJOA patients were validated using qRT-PCR. The genes were selected based on the significant differential expression in the RNA-seq dataset (GSE289871) and their relevance to TMJOA pathogenesis. (**A**) CRIP1: Significantly upregulated in TMJOA (*p* = 0.0342), (**B**) PPA1: Significantly upregulated in TMJOA (*p* = 0.0362), (**C**) TARS1: Significantly upregulated in TMJOA (*p* = 0.0293), and (**D**) GCLC: Increased expression in TMJOA but not statistically significant. Statistical analysis was performed using an unpaired *t*-test with unequal variance. Data are presented as mean ± SD. * *p* < 0.05.

Four genes identified from the clinical RNA-seq dataset (GSE289871), *CRIP1*, *PPA1*, *TARS1* and *GCLC*, were selected for validation using the same criteria. These genes were frequently enriched in pathways related to TMJOA pathogenesis, significantly upregulated in TMJOA patients based on RNA-seq data, and were biologically relevant to oxidative stress, metabolism, or immune regulation according to the existing literature. Validation by qRT-PCR revealed that *CRIP1*, *PPA1* and *TARS1* were significantly elevated in TMJOA patients (*p* = 0.0342, 0.0362, and 0.0293; Figure 5A–C). GCLC also showed increased expression but did not reach statistical significance (Figure 5D). After the Benjamini–Hochberg correction for multiple testing across the qRT-PCR validation panel has been performed, PPA1 approached the threshold of statistical significance (FDR-adjusted *p*-value = 0.05), whereas CRIP1 (FDR-adjusted *p*-value = 0.07) and TARS1 (FDR-adjusted *p*-value = 0.12) did not retain statistical significance following adjustment. GCLC showed an increased expression but did not reach nominal statistical significance (FDR-adjusted *p*-value = 0.23).

**Figure 6:**
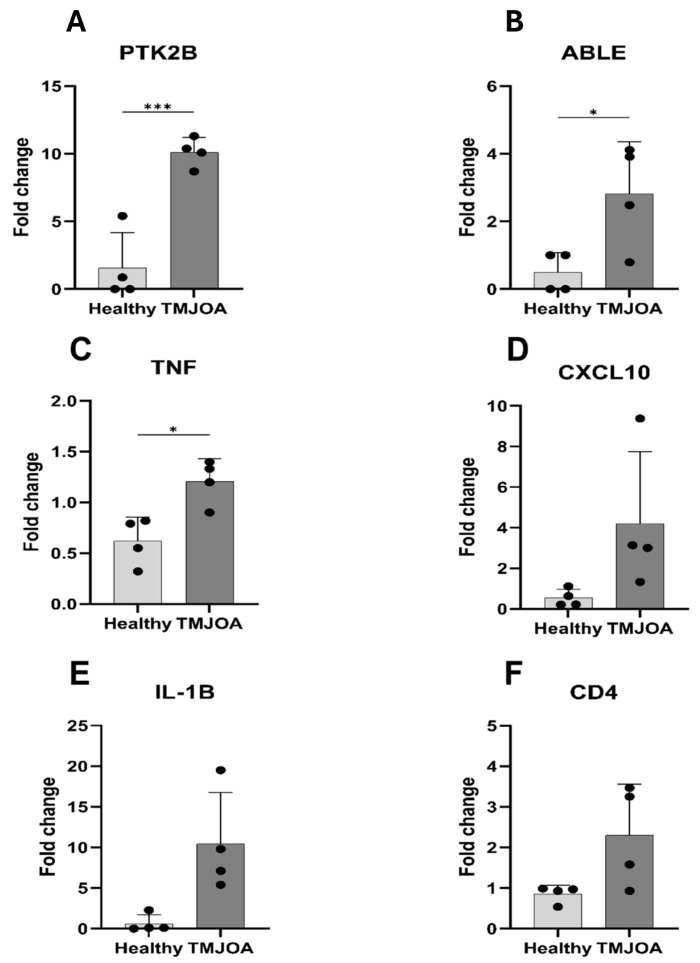
qRT-PCR validation of in silico-identified genes in the dataset (GSE205389). Bar graphs comparing the expression levels of six genes between the healthy control group and the TMJOA group, based on qRT-PCR analysis. These genes were selected from the in silico dataset (GSE205389) for validation due to their frequent enrichment in disease-relevant pathways and strong differential expression. (**A**–**C**) PTK2B, ABL1 and TNF were significantly upregulated in TMJOA patients with *p*-values of 0.0009, 0.0300, and 0.0107, respectively. (**D**–**F**) IL-1B (*p* = 0.0218) showed a significant upregulation, while CXCL10 (*p* = 0.0855) and CD4 (*p* = 0.0618) showed increased expression that did not reach statistical significance. Statistical analysis was performed using an unpaired *t*-test with unequal variance. A *p*-value ≤ 0.05 was considered statistically significant. * indicates *p* < 0.05 and *** indicates *p* < 0.001. Data are presented as mean ± SD.

Similarly, a total of six genes from the in silico dataset (GSE205389) were selected for validation: *PTK2B*, *ABL1*, *TNF*, *IL-1B*, *CXCL10* and *CD4*. The qRT-PCR results confirmed that *PTK2B*, *ABL1*, *TNF* and *IL-1B* were significantly upregulated in TMJOA patients compared to controls (*p*-values: 0.0009, 0.0300, 0.0107, and 0.0218, respectively; Figure 6A–C). After the Benjamini–Hochberg correction for multiple testing across the qRT-PCR validation panel has been performed, PTK2B (FDR = 0.01), TNF (FDR = 0.03), and IL-1B (FDR = 0.04) remained statistically significant, whereas ABL1 reached the threshold of statistical significance (FDR = 0.05). Although *CXCL10* and *CD4* were also elevated in the TMJOA group, their expression did not reach nominal statistical significance (*p* = 0.0855 and 0.0618; Figure 6D–F). These findings support the utility of these genes as candidate salivary biomarkers for TMJOA and further confirm their involvement in key pathological pathways of the disease.

## 3. Discussion

This study presents a comprehensive transcriptomic investigation identifying candidate salivary biomarkers for TMJOA using saliva-derived RNA. Transcriptomic profiling of biofluids such as saliva enables the capture of systemic and upstream regulatory molecular events that reflect global disease-associated networks, even when the primary affected tissue is not directly accessible. Importantly, transcriptomic approaches are less influenced by post-translational modifications and compensatory feedback mechanisms that can obscure disease-relevant signals in proteomic analyses. This strategy has been successfully applied in malignancies such as prostate and colorectal cancer, where circulating RNA signatures support disease risk stratification, early detection, and molecular disease association that is independent of direct tissue accessibility [17,19,20].

In the context of joint and inflammatory disorders, saliva has increasingly been recognized as a reliable surrogate biofluid reflecting systemic immune activation and metabolic dysregulation [10,11,13,14,15,16]. Several studies have demonstrated strong correlations between salivary and serum inflammatory mediators, including cytokines, oxidative stress markers, and immune-related transcripts [21,22]. While saliva is influenced by local oral conditions [8,9], strict exclusion criteria for periodontal disease, active infection, and inflammatory comorbidities were applied in this study to minimize confounding effects. Together, these features support saliva as a biologically relevant and clinically practical platform for non-invasive molecular profiling in TMJOA.

Through the integration of saliva-derived RNA-seq with in silico data and validation via qRT-PCR, we observed an upregulation of *CRIP1*, *PPA1* and *TARS1* in TMJOA patients. These genes were associated with inflammation, oxidative stress, and metabolic dysfunction, which are hallmarks of osteoarthritic pathology.

*CRIP1*, a cysteine-rich intestinal protein with a double zinc finger motif, has been linked to inflammatory conditions such as periodontitis and osteonecrosis. Its upregulation in TMJOA suggests a potential role in mediating cartilage or synovial membrane inflammation [23,24]. Similarly, *PPA1*, a key enzyme in pyrophosphate hydrolysis, regulates phosphate homeostasis, which is critical for chondrocyte function [25,26,27]. *TGF-β* is known to elevate extracellular PPi in chondrocytes via PC-1, a mechanism affected by aging and mitochondrial metabolism. Elevated *PPA1* may thus reflect altered energy signaling in TMJOA cartilage [28].

*TARS1*, encoding threonyl-tRNA synthetase, is increasingly recognized for its role in immune modulation. It promotes dendritic cell maturation and *IL-12* secretion, which polarizes CD4+ T-cells toward a Th1 response—an axis also implicated in osteoarthritic inflammation [29]. In angiogenic conditions, *TARS1* can be secreted by endothelial cells in response to VEGF and TNF-α, contributing to local vascular changes in the TMJOA tissue [30].

In our RNA-seq analysis, GCLC was identified as an altered gene at the nominal threshold. *GCLC*, also known as gamma-glutamylcysteine synthetase, is the first and rate-limiting enzyme in glutathione (GSH) biosynthesis. During the inflammatory response of macrophages, GCLC expression is downregulated, contributing to GSH depletion and oxidative stress. The underlying mechanism involves increased mRNA degradation and enhanced protein degradation that are mediated by caspase-5, as shown in response to LPS stimulation. A supplementation with γ-glutamylcysteine (γ-GC), a direct product of GCLC activity, was found to restore intracellular GSH levels and mitigate LPS-induced inflammation [31]. *GCLC* may be involved in the oxidative stress component of TMJOA pathophysiology.

qRT-PCR validation also showed an upregulation of *PTK2B* and *ABL1* in TMJOA samples. PTK2B, a calcium-dependent kinase, is activated downstream of an intracellular calcium influx and regulates endothelial integrity via the phosphorylation of VE-PTP. Its role in TMJOA may extend to vascular remodeling and inflammatory signaling [32,33]. ABL1, a proto-oncogene with stress response and DNA-repairing functions, may mediate synovial fibroblast proliferation and matrix remodeling [34].

Inflammatory cytokines were central to our findings. TNF-α, which was upregulated in TMJOA, binds TNFR1 to activate NF-κB and MAPK pathways, promoting the production of prostaglandins, COX-2, IL-6, and MMPs. It also suppresses Th1/NK cells while enhancing effector-T cell activation. IL-1β, another upregulated cytokine, inhibits type II collagen synthesis and increases MMPs and ADAMTS, driving cartilage degradation. Its activation of chemokines like IL-8, MCP-1, and RANTES supports further immune cell infiltration, perpetuating the inflammatory cycle [35,36,37,38].

Additional cytokines such as IL-6 and IL-17, while not directly measured in our qRT-PCR panel, are known contributors to TMJOA. IL-6 promotes cytotoxic T-cell and NK cell differentiation, while IL-17 enhances synovial invasion, cartilage degradation, and angiogenesis, findings that are consistent with the enriched pathways identified here [39,40,41].

Enrichment analysis of DEGs using GSEA and Metascape identified key TMJOA-relevant pathways, including actin filament organization, osteoclast differentiation, immune cell migration, and amino acid metabolism. Notably, actin cytoskeleton remodeling is essential for the chondrocyte shape, mechano-transduction, and matrix production. Osteoclast differentiation pathways also explain the subchondral bone erosion commonly seen in TMJOA [42,43].

Of particular interest, we found 743 DEGs common to both in silico and experimental datasets, supporting consistency between the datasets. These shared genes enriched pathways, including neutrophil degranulation, chemokine signaling, and cellular responses to stress, are all implicated in joint degeneration. This study is not without limitations. The relatively small sample size, although statistically powered for initial discovery, limits the generalizability of the results. In addition, the cross-sectional design of the study does not allow for the assessment of temporal changes, disease progression, or the prognostic value of the identified biomarkers. Furthermore, protein-level confirmation and mechanistic studies are needed to translate these transcriptomic signatures into clinical biomarkers or therapeutic targets. The identified biomarkers are currently specific to TMJOA and cannot be generalized to the osteoarthritis that affects other anatomical joints. Considering the distinct anatomical configuration, functional biomechanics, and molecular pathophysiology of the TMJ, the extrapolation of these findings to systemic or generalized osteoarthritis remains unsupported at this stage. In summary, this study identifies a novel panel of genes, *CRIP1*, *PPA1*, *TARS1* and *GCLC*, along with cytokines *TNF* and *IL-1β* and signaling mediators *PTK2B* and *ABL1*, as candidate salivary biomarkers in TMJOA. These findings highlight the key inflammatory and metabolic pathways driving disease progression and offer a foundation for future diagnostic and therapeutic strategies tailored to the early and non-invasive detection of TMJOA.

It is important to note that this study was designed as an exploratory hypothesis-generating proof-of-concept investigation to identify candidate salivary transcriptomic biomarkers associated with TMJOA rather than to establish definitive diagnostic markers. To ensure robustness, differential expression analysis was complemented by experimental validation and pathway-level enrichment analyses, thereby supporting biological interpretation. Although qRT-PCR validation confirmed technical concordance with RNA-seq results, validation in independent and larger clinical cohorts will be essential to establish biological robustness and clinical generalizability of these candidate biomarkers as well as to enable more advanced statistical modeling, multiple-testing correction, and formal assessment of diagnostic performance. While larger cohorts and prospective validation will be required for clinical translation, the present work provides an initial molecular framework for non-invasive TMJOA biomarker development.

Furthermore, while this study focused on a clinically defined cohort of TMJOA patients aged 40–55 years, the biological pathways identified, including inflammatory signaling, immune activation, cytoskeletal remodeling and metabolic stress responses, are fundamental mechanisms that are implicated in osteoarthritis and joint degeneration across age groups and populations. Therefore, although the current candidate biomarker signature was derived from a specific demographic group, it is likely reflective of broader disease-related molecular processes. Nevertheless, future studies involving larger, age-diverse cohorts with balanced sex distribution will be essential to validate the generalizability and clinical utility of these candidate salivary biomarkers.

## 4. Materials and Methods

### 4.1. Overview of Study Design and Analytical Workflow

This study followed a multi-phase design combining clinical sample analysis with in silico transcriptomic analysis to identify candidate salivary biomarkers that are associated with TMJOA. RNA extracted from clinical saliva samples (GSE289871) and publicly available RNA-seq data (GSE205389) were independently analyzed using DESeq2 to identify DEGs. Significant DEGs from both datasets were subjected to Gene Set Enrichment Analysis (GSEA) using C5 and C7 gene sets to uncover enriched biological and immunological pathways. Gene frequency analysis was performed to identify the most recurrent genes across enriched pathways, as previously described [44,45]. The top candidates were then validated using qRT-PCR in the clinical cohort. Additionally, Metascape was used to confirm shared pathways across datasets. The complete workflow is outlined in Supplementary Figure S1.

### 4.2. Ethical Approval

The study was approved by the University of Sharjah Research Ethics Committee (REC-23-10-17-01-PG) on 11 October 2023.

### 4.3. Patient Samples

Sample size for RNA-seq analysis was calculated using the “power.t.test” function in R (version 4.3.2), based on parameters from Wei et al. [46] with effect size = 2.6, standard deviation = 1.5, significance level = 0.05 and power = 0.9. This yielded a minimum of 8 samples per group. Biomarker validation was conducted using a cohort of 8 TMJOA patients and 8 healthy controls. Due to the limited cohort size, stratified analyses by sex were not performed; however, this can be addressed in future studies with larger patient populations.

Saliva samples were collected from patients attending the Oral Diagnosis and Urgent Care Clinic at the University Dental Hospital, Sharjah (UAE), between November 2023 and May 2024 using a stratified random sampling method. Participants were categorized into two subgroups, TMJOA patients and healthy individuals, based on clinical diagnosis and pre-defined inclusion and exclusion criteria.

Specifically, patients aged 18–60 years presenting with joint sounds, joint pain, deviation in mouth opening, or functional disabilities were included in the TMJOA group. Healthy individuals in the same age range without TMD served as controls. Exclusion criteria included prior TMD treatment, oral lesions including ulcers and periodontal diseases, and pregnancy. Additional exclusion criteria included systemic inflammatory diseases, autoimmune disorders, metabolic bone diseases, active infections, and use of immunosuppressive or anti-inflammatory medications unrelated to TMJOA, to minimize potential confounding effects on salivary transcriptomic profiles. A total of 16 participants were enrolled: 8 TMJOA patients confirmed by radiological findings and 8 healthy controls, with a mean age of 46.9 years. The TMJOA group consisted of 4 females and 4 males, while the control group included 4 females and 4 males. All participants provided written informed consent before participation.

Diagnostic assessment followed the DC/TMD criteria, including clinical examination, 36 standardized questionnaires and routine radiographic examination. Patients classified as Di III on the Helkimos clinical dysfunction index (Di) were referred for a cone beam computed tomography (CBCT) examination to confirm the TMJOA diagnosis [42]. Supplementary Table S1 shows the patient demographics and disease staging based on radiological assessment.

### 4.4. Sample Collection

Saliva samples (2–3 mL) were collected from the participants without stimulation, at least 3–4 h after food intake. Each sample was immediately mixed with 1 mL of RNAlater^TM^ stabilization solution (AM7021, Thermo Fisher Scientific, Baltics UAB, Vilnius, Lithuania).

### 4.5. RNA Extraction

Total RNAs were extracted from 700 μL of each saliva sample using the RNeasy kit (Qiagen, Hilden, Germany) following the manufacturer’s recommendations. RNA concentration and purity were assessed using a NanoDrop^TM^ 2000 spectrophotometer (ThermoFisher Scientific, Waltham, MA, USA). All RNA samples were stored at −80 °C until further analysis [19,47].

### 4.6. Whole Transcriptome Sequencing of RNA Extracted from Saliva Samples

Genomic DNA was removed from the RNA samples using Turbo DNase (ThermoFisher Scientific, USA). Whole transcriptome sequencing was then performed on 8 samples (healthy group) and 8 samples (TMJOA group) (Supplementary Table S1) using targeted RNA-seq with the Ion AmpliSeq Whole Transcriptome Human Gene Expression kit (ThermoFisher Scientific, USA). cDNA was synthesized using the SuperScript^TM^ VILO cDNA Synthesis kit (ThermoFisher Scientific, USA) and amplified with Ion AmpliSeq human gene expression core panel primers. Amplicons (~200 bp) were enzymatically sheared, then they were ligated with adapters and unique barcodes and purified using Agencourt AMPure XP Beads (Beckman Coulter, Brea, CA, USA). Libraries were quantified with the Ion Library TaqMan^TM^ quantitation kit (Applied Biosystems, Waltham, MA, USA), diluted to 100 pM and pooled equally across 16 individual samples. The diluted libraries were then amplified and enriched using the Ion Chef System (ThermoFisher Scientific) according to the manufacturer’s instructions. Sequencing was carried out on an Ion S5 XL Semiconductor sequencer (ThermoFisher Scientific) using Ion 540 Chips.

### 4.7. RNA Sequencing Data Analysis of Saliva-Derived Samples GSE289871

RNA-seq data that were obtained from 16 clinical saliva samples were analyzed using the Ion Torrent Software Suite (Version 5.4). Raw sequencing reads were aligned to the human genome reference sequence (hg19/GRCh37) using the Torrent Mapping Alignment Program (TMAP), which applies a two-stage mapping method combining BWA-short, BWA-long, SSAHA [42], Super-Maximal Exact Matching [39] and the Smith–Waterman algorithm [43]. The raw read counts were used as input for differential expression analysis using DESeq2 R/Bioconductor, which performs internal normalization based on size factors. Fragments per kilobase million (FPKM) values were calculated separately and used for data visualization and exploratory analyses, including principal component analysis (PCA) in the R statistical program (version 4.3.2). Genes showing fewer than 10 read counts were excluded. DEGs were required to meet a statistical significance threshold of *p* < 0.05 and selected to retain biologically relevant candidates in this exploratory, hypothesis-generating proof-of-concept cohort. To minimize false-positive findings, robustness was ensured through experimental validation and pathway-level enrichment analyses by incorporating false discovery rate (FDR) control. Sequencing data was uploaded to the GEO database under accession number GSE289871. The custom R scripts used for analysis are available upon request.

### 4.8. In Silico Data Analysis of Publicly Available Transcriptomic Dataset GSE205389

In parallel with our clinical RNA-seq analysis, an in silico investigation was conducted using a publicly available transcriptomic dataset retrieved from the Gene Expression Omnibus (GEO) under accession number GSE205389 (accessed on 15 July 2024), which included TMJOA patients (*n* = 5) and healthy controls (*n* = 5). The publicly available count matrix was used to perform differential gene expression analysis using DESeq2. Differentially expressed genes were identified at a nominal threshold of *p* < 0.05 and were considered exploratory candidates.

### 4.9. Gene Set Enrichment Analysis (GSEA) from the Two Datasets GSE289871 and GSE205389

The filtered DEGs from both RNA-seq datasets (our clinical cohort GSE289871) and the publicly available dataset (GSE205389) were then subjected to GSEA to identify pathways associated with TMJOA. Approximately 90,000 annotated pathways from the Broad Institute’s database (https://www.gsea-msigdb.org, accessed on 10 October 2024), along with custom-defined pathways, were used. Standard and absolute GSEA (absGSEA) were performed utilizing the MSigDB with particular emphasis on the C5 (Gene Ontology) and C7 (immunological signatures) collections due to their relevance to molecular, biological, inflammatory and immune-related processes. The pathways were considered significantly enriched if they met a nominal *p*-value < 0.05 and an FDR < 0.25. Most altered DEGs with log_2_ fold change (log_2_FC) >±1.5 appearing in multiple enriched pathways and relevant to TMJOA pathogenesis were prioritized for downstream analysis. This cutoff corresponds to an approximate ≥2.8-fold change in gene expression, representing a biologically stringent effect size. In addition, given the inherent variability of saliva-derived samples, an additional expression-level criterion was imposed: candidate genes were required to exhibit an average raw read count greater than 100 in the first group (healthy or TMJOA patients) and greater than 30 in the second group (healthy or TMJOA patients).

### 4.10. Quantitative Real-Time Polymerase Chain Reaction (qRT-PCR)

Highly expressed and most alerted candidate biomarkers appearing in more than three pathways relevant to TMJOA pathogenesis from both datasets were selected for further validation using qRT-PCR. Complementary DNA (cDNA) was synthesized from the total RNA extracted from the saliva samples of healthy and TMJOA patients using the High-Capacity cDNA Reverse Transcription Kit (Applied Biosystems, Waltham, MA, USA) in accordance with the manufacturer’s instructions. qRT-PCR was performed using the QuantStudio3 Real-Time PCR thermal cycler (Applied Biosystems, Waltham, MA, USA) and Maxima SYBR Green/ROX qPCR MasterMix (Thermo Scientific, USA). Each sample was analyzed in three technical replicates. The primers set used in qRT-PCR runs is listed in the Supplementary Table S2. Glyceraldehyde 3-phosphate dehydrogenase (GAPDH) was used as the housekeeping gene for normalization. Relative gene expression was calculated using the 2^−(ΔΔCt)^ method.

### 4.11. Comparative Enrichment Analysis Using Metascape

Functional enrichment and pathway analysis were performed on the commonly altered DEGs between the two datasets: GSE289871 and GSE205389 using the Metascape annotation tool (v3.5.20260201, http://metascape.org, accessed on 21 December 2024) in order to complement the previous enrichment results obtained via GSEA. The commonly altered genes were identified using the InteractiVenn tool (http://www.interactivenn.net, accessed on 9 January 2025) to visualize and verify the shared genes between the two datasets.

### 4.12. Statistical Data Analysis

For qRT-PCR analysis, statistical comparisons between TMJOA patients and healthy controls were performed using an unpaired *t*-test assuming unequal variances (Graph pad Prism 8.4). To address multiple testing in the qRT-PCR validation panel (10 genes), Benjamini–Hochberg FDR correction was applied. The results are presented as mean ± standard deviation (SD).

## 5. Conclusions

In this proof-of-concept study, CRIP1, PPA1, TARS1 and GCLC were identified as candidate genes that were potentially involved in the inflammatory processes and in the progression of TMJOA, supporting their role as candidate salivary biomarkers. These targets warrant further investigation to determine their possible diagnostic and therapeutic relevance. Given the study’s focus on an Arab clinical cohort, the findings offer a valuable foundation for future diagnostic and therapeutic approaches that are tailored to this population. However, the relatively small sample size limits the generalizability of the results. The larger multi-center studies involving diverse populations are needed to validate these candidate biomarkers and assess their broader clinical relevance.

## Data Availability

The data underlying this article are available in the article and in its online Supplementary Materials. The sequencing data are uploaded to the GEO database under the accession number GSE289871. The custom R scripts that were used for analysis are available upon request.

## Supplementary Materials

The following supporting information can be downloaded at https://www.mdpi.com/article/10.3390/ijms27062727/s1.
