# Supplementary material for: Whole Transcriptomic Analysis Identifies Candidate Biomarkers from Saliva of Temporomandibular Joint Osteoarthritis Patients
Source: Int J Mol Sci. 2026 Mar 17;27(6):2727. doi: 10.3390/ijms27062727 (PMC13026090; doi:10.3390/ijms27062727)
Supplement: Supplementary file 1 [file ijms-27-02727-s001.zip › ijms-4137818-supplementary.pdf]

Whole Transcriptomic Analysis Identifies Candidate Biomarkers from Saliva of Temporomandibular Joint osteoarthritis patients

Nawal Alketbi[1,*], Alaa Muayad Altaie[2,3], Reem Sami Alhamidi[2], Ayesha Yusuf Phansupkar[2], Alaa Mohamed Hamad[4], Mohamed Haider[2,5], Rania Harati[2,6], Kathrin Kalies[7], Wael Talaat[1,2,†], Rifat Hamoudi[2,3,8,9,10,*,†]

Supplementary Figures

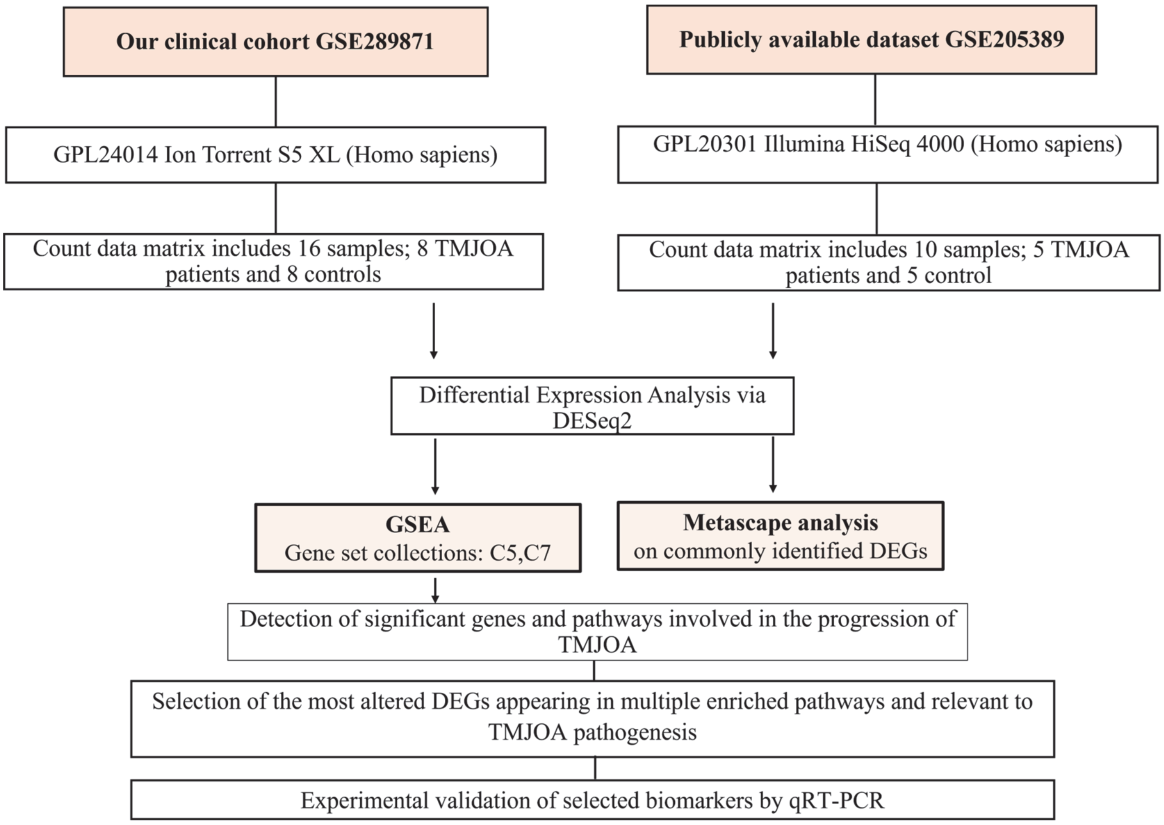

Figure S1. Overview of the Study Design and Bioinformatics Workflow for TMJOA Biomarker Discovery.
This flowchart illustrates the integrated analytical approach used to identify salivary biomarkers in TMJOA. RNA-seq data from our clinical cohort (GSE289871; 8 TMJOA patients and 8 healthy controls) and a publicly available *in silico* dataset (GSE205389; 5 TMJOA patients and 5 controls) were independently analysed using DESeq2 to identify DEGs. Subsequent GSEA was performed using C5 (biological processes) and C7 (immunologic signatures) gene collections. Common DEGs between datasets were further analysed using Metascape for functional clustering and pathway validation. The most frequently altered DEGs involved in multiple enriched pathways relevant to the diseases were prioritized and experimentally validated via qRT-PCR in clinical saliva samples.

Supplementary Tables

Table S1. Clinical characteristics and radiological assessment of the included patients. Samples were used for RNAseq (GSE289871).

Patient	Age	Sex	Radiological assessment
Sample 1	42	Female	OPG- Healthy
Sample 2	45	Female	OPG- Healthy
Sample 3	39	Female	OPG- Healthy
Sample 4	58	Female	OPG- Healthy
Sample 5	49	Male	OPG- Healthy
Sample 6	47	Male	OPG- Healthy
Sample 7	47	Male	OPG- Healthy
Sample 8	52	Male	OPG- Healthy
Sample 9	59	Female	CBCT – TMJOA
Sample 10	36	Male	CBCT – TMJOA
Sample 11	60	Male	CBCT – TMJOA
Sample 12	41	Male	CBCT - TMJOA
Sample 13	36	Female	CBCT - TMJOA
Sample 14	48	Male	CBCT - TMJOA
Sample 15	34	Female	CBCT - TMJOA
Sample 16	49	Female	CBCT - TMJOA

Table S2. Primers set used for qRT-PCR

Gene	Accession number	Forward primer (5′~3′)	Reverse primer (5′~3′)
PTK2B	NM_173176.3	CATTAACTTCCGACGCTTCACG	GATGACATCCTTGTTCTCCAGC
ABL1	NM_007313.3	GTGGAGATAACACTCTAAGCAT	CCATTTTTGGTTTGGGCTTCAC
TNF	NM_000594.4	GGCGCCACCACGCTCTTCC	TGGGCCAGAGGGCTGATTAG
CXCL10	NM_001565.4	GTACGCTGTACCTGCATCAG	GCAATGATCTCAACACGTGG
IL-1B	NM_000576.3	GATGATAAGCCCACTCTACAGC	GAACTGGGCAGACTCAAATTCC
CD4	NM_001195017.3	CCTCCTGCTTTTCATTGGGCTAG	TGAGGACACTGGCAGGTCTTCT
CRIP1	NM_001311.5	CAAGTGTCCCAAGTGCAACAA	CCCACATTTCTCGCACTTCAG
PPA1	NM_021129.4	GAAGGTATAAGGTTCCTGATG	CTTTCCAATGGTCATGAGTGC
TARS1	NM_152295.5	CATATTCTGTGCCATGGAGC	TCCGGGCGAGTAGAAAGGTT
GCLC	NM_001498.4	ATCCTCCAGTTCCTGCACAT	GACAGGACCAACCGGACTTT

Table S3. The list of enriched pathways sets in C5 and C7 in our clinical RNA-seq dataset (GSE289871)

	C5
1.	GOBP_AMINO_ACID_METABOLIC_PROCESS
2.	GOBP_ALPHA_AMINO_ACID_METABOLIC_PROCESS
3.	GOBP_VITAMIN_METABOLIC_PROCESS
4.	HP_ABNORMAL_CIRCULATING_NITROGEN_COMPOUND_CONCENTRATION
5.	GOBP_MITOTIC_NUCLEAR_DIVISION
6.	HP_POOR_SUCK
7.	GOMF_HEAT_SHOCK_PROTEIN_BINDING
	C7
1.	ZAK_PBMC_MRKAD5_HIV_1_GAG_POL_NEF_AGE_20_50YO_CORRELATED_WITH_CD8_T_CELL_RESPONSE_3DY_POSITIVE
2.	GSE21546_UNSTIM_VS_ANTI_CD3_STIM_DP_THYMOCYTES_DN
3.	GSE10240_CTRL_VS_IL17_STIM_PRIMARY_BRONCHIAL_EPITHELIAL_CELLS_UP
4.	GSE36078_UNTREATED_VS_AD5_T425A_HEXON_INF_IL1R_KO_MOUSE_LUNG_DC_UP
5.	THAKAR_PBMC_INACTIVATED_INFLUENZA_AGE_70PLS_NONRESPONDER_2DY_UP
6.	GSE45365_HEALTHY_VS_MCMV_INFECTION_CD11B_DC_DN
7.	GSE2770_TGFB_AND_IL4_VS_TGFB_AND_IL12_TREATED_ACT_CD4_TCELL_6H_UP
8.	GSE20198_UNTREATED_VS_IL12_TREATED_ACT_CD4_TCELL_DN
9.	GSE17580_UNINFECTED_VS_S_MANSONI_INF_TEFF_DN
10.	GSE40068_BCL6_POS_VS_NEG_CXCR5_POS_TFH_DN
11.	GSE17721_0.5H_VS_12H_POLYIC_BMDC_UP
12.	GSE37532_WT_VS_PPARG_KO_LN_TCONV_DN
13.	GSE30962_ACUTE_VS_CHRONIC_LCMV_SECONDARY_INF_CD8_TCELL_DN
14.	GSE25088_CTRL_VS_IL4_STIM_STAT6_KO_MACROPHAGE_UP
15.	GSE9960_GRAM_NEG_VS_GRAM_NEG_AND_POS_SEPSIS_PBMC_DN
16.	ZAK_PBMC_MRKAD5_HIV_1_GAG_POL_NEF_AGE_20_50YO_CORRELATED_WITH_CD8_T_CELL_RESPONSE_3DY_POSITIVE

Table S4. The list of the enriched pathways set in both C5 and C7 in the publicly available *in silico* dataset (GSE205389)

	C5
1.	GO_ACTIN_MONOMER_BINDING
2.	GO_ACTIN_BINDING
3.	GO_CHAPERONE_BINDING
4.	GO_ACTIN_FILAMENT_BINDING
5.	GO_CYTOKINE_BINDING
6.	GO_PROTEIN_HOMODIMERIZATION_ACTIVITY
7.	GO_SH3_DOMAIN_BINDING
8.	GO_AMIDE_BINDING
9.	GO_COLLAGEN_BINDING
10.	GO_CYTOSKELETAL_PROTEIN_BINDING
11.	GO_MOTOR_ACTIVITY
12.	GO_PROTEIN_DIMERIZATION_ACTIVITY
13.	GO_PROTEIN_CONTAINING_COMPLEX_BINDING
14.	GO_PROTEIN_PHOSPHATASE_BINDING
15.	GO_PEPTIDE_BINDING
16.	GO_ATPASE_ACTIVITY_COUPLED
17.	GO_ACTIN_MONOMER_BINDING
18.	GO_POSITIVE_REGULATION_OF_CALCIUM_ION_TRANSPORT
19.	GO_ANATOMICAL_STRUCTURE_FORMATION_INVOLVED_IN_MORPHOGENESIS
20.	GO_REGULATION_OF_OSTEOCLAST_DIFFERENTIATION
21.	GO_REGULATION_OF_CALCIUM_MEDIATED_SIGNALING
22.	GO_SYNAPSE_ORGANIZATION
23.	GO_RESPONSE_TO_PEPTIDE
24.	GO_ACTIN_FILAMENT_BASED_PROCESS
25.	GO_CYTOSKELETON_ORGANIZATION
26.	GO_POSITIVE_REGULATION_OF_TRANSPORTER_ACTIVITY
27.	GO_REGULATION_OF_CELLULAR_COMPONENT_SIZE
28.	GO_SECOND_MESSENGER_MEDIATED_SIGNALING
29.	GO_REGULATION_OF_PROTEIN_POLYMERIZATION
30.	GO_ACTIN_POLYMERIZATION_OR_DEPOLYMERIZATION
31.	GO_CALCIUM_MEDIATED_SIGNALING

32.	GO_REGULATION_OF_ACTIN_FILAMENT_BASED_PROCESS
33.	GO_POSITIVE_REGULATION_OF_RELEASE_OF_SEQUESTERED_CALCIUM_ION_INTO_CYTOSOL
34.	GO_REGULATION_OF_ANATOMICAL_STRUCTURE_SIZE
35.	GO_POSITIVE_REGULATION_OF_CALCIUM_MEDIATED_SIGNALING
36.	GO_CELLULAR_RESPONSE_TO_HORMONE_STIMULUS
37.	GO_PROTEIN_POLYMERIZATION
38.	GO_POSITIVE_REGULATION_OF_ION_TRANSPORT
39.	GO_CYTOSOLIC_CALCIUM_ION_TRANSPORT
40.	GO_POSITIVE_REGULATION_OF_CYTOSKELETON_ORGANIZATION
41.	GO_REGULATION_OF_METAL_ION_TRANSPORT
42.	GO_REGULATION_OF_CALCIUM_ION_TRANSPORT
43.	GO_RESPONSE_TO_HORMONE
44.	GO_ACTOMYOSIN_STRUCTURE_ORGANIZATION
45.	GO_REGULATION_OF_CALCIUM_ION_TRANSPORT_INTO_CYTOSOL
46.	GO_ORGANELLE_ASSEMBLY
47.	GO_FEMALE_SEX_DIFFERENTIATION
48.	GO_CELLULAR_RESPONSE_TO_NITROGEN_COMPOUND
49.	GO_POSITIVE_REGULATION_OF_CALCIUM_ION_TRANSPORT_INTO_CYTOSOL
50.	GO_RESPONSE_TO_FATTY_ACID
51.	GO_NEGATIVE_REGULATION_OF_I_KAPPAB_KINASE_NF_KAPPAB_SIGNALING
52.	GO_ACTIN_FILAMENT_POLYMERIZATION
53.	GO_REGULATION_OF_CYTOSKELETON_ORGANIZATION
54.	GO_NEGATIVE_REGULATION_OF_CALCIUM_ION_TRANSPORT
55.	GO_MULTICELLULAR_ORGANISMAL_HOMEOSTASIS
56.	GO_CELLULAR_COMPONENT_MORPHOGENESIS
57.	GO_REGULATION_OF_ATPASE_ACTIVITY
58.	GO_POSITIVE_REGULATION_OF_ACTIN_FILAMENT_POLYMERIZATION
59.	GO_ACTIN_FILAMENT_ORGANIZATION
60.	GO_REGULATION_OF_RELEASE_OF_SEQUESTERED_CALCIUM_ION_INTO_CYTOSOL
61.	GO_ACTIN_NUCLEATION
62.	GO_CELLULAR_COMPONENT_DISASSEMBLY
63.	GO_POSITIVE_REGULATION_OF_PROTEIN_POLYMERIZATION
64.	GO_MUSCLE_CELL_MIGRATION
65.	GO_RESPONSE_TO_MECHANICAL_STIMULUS
66.	GO_CIRCULATORY_SYSTEM_DEVELOPMENT
67.	GO_NEGATIVE_REGULATION_OF_INTRACELLULAR_TRANSPORT
68.	GO_DIVALENT_INORGANIC_CATION_TRANSPORT
69.	GO_SMOOTH_MUSCLE_CELL_MIGRATION
70.	GO_PROTEIN_CONTAINING_COMPLEX_DISASSEMBLY
71.	GO_RESPONSE_TO_PEPTIDE_HORMONE
72.	GO_SEQUESTERING_OF_CALCIUM_ION
73.	GO_SUPRAMOLECULAR_FIBER_ORGANIZATION
74.	GO_CALCIUM_ION_TRANSMEMBRANE_IMPORT_INTO_CYTOSOL
75.	GO_REGULATION_OF_ACTIN_FILAMENT_ORGANIZATION
76.	GO_REGULATION_OF_SUPRAMOLECULAR_FIBER_ORGANIZATION
77.	GO_POSITIVE_REGULATION_OF_SUPRAMOLECULAR_FIBER_ORGANIZATION
C7	
1.	GSE20715_WT_VS_TLR4_KO_48H_OZONE_LUNG_UP
2.	GSE339_CD4POS_VS_CD4CD8DN_DC_IN_CULTURE_UP
3.	GSE32901_NAIVE_VS_TH17_NEG_CD4_TCELL_DN
4.	GSE42021_CD24INT_TREG_VS_CD24INT_TCONV_THYMUS_DN

Table S5. Top 20 genes obtained from the gene frequency analysis in TMJOA vs control based on absGSEA results from both our clinical RNA-seq dataset (GSE289871) and the publicly available dataset (GSE205389)

GSE289871	GSE205389
GCLC	*PTK2B*
PSAT1	*ABL1*
SHMT2	*CORO1A*
ATPAF2	*ACTN2*
DDAH1	*ICAM1*
ENOPH1	*CASQ1*
HIBADH	*CCR7*
HSPA1A	*PFN1*
LGSN	*LMOD1*
MMAA	*CAV3*
ODC1	*LMOD3*
PCBD2	*LMOD2*
ALDH1A1	*TRIM27*
ALG5	*DLG1*
ALG9	*PFN2*
CEP97	*PYCARD*
GATM	*CCL21*
HNMT	*NCKAP1L*
HSPA8	*CORO1B*
IQCG	*GRB2*
